# Comparing the Effects of Rhythm-Based Music Training and Pitch-Based Music Training on Executive Functions in Preschoolers

**DOI:** 10.3389/fnint.2019.00041

**Published:** 2019-08-27

**Authors:** Ulrike Frischen, Gudrun Schwarzer, Franziska Degé

**Affiliations:** Department of Developmental Psychology, Faculty of Psychology and Sports Science, Justus-Liebig-University, Giessen, Germany

**Keywords:** music training, rhythm, pitch, executive functions, inhibition, preschoolers

## Abstract

Previous research has indicated the beneficial effects of music training on executive functions (EFs) in children. However, researchers have not clearly determined which component of music training produces these beneficial effects or whether different components exert different effects on EFs. In the present study, we examined the impact of rhythm-based music training compared to pitch-based music training and sports training as a control on EFs in preschoolers. Children aged between 5 and 6 years (*N* = 76) were randomly assigned to one of the three training groups and received training in small groups three times a week for 20 min in German kindergartens. Before and after training, children completed tests designed to assess inhibition, set-shifting, and visuospatial working memory. Parental education, family income, personality, and IQ served as control variables. We observed a significant training group × time interaction for the measure of inhibition. Children from the rhythm group exhibited significant improvements in inhibition from pre- to post-tests (*d*_RM_ = 0.56), whereas children from the other groups did not. Furthermore, children from the rhythm group significantly differed from the sports control group at post-test (*d*_corr_ = 0.82). Concerning the measures of set-shifting and visuospatial working memory, the descriptive data revealed similar results; however, we did not observe significant training group × time interactions. Based on our findings, rhythm-based music training specifically enhances inhibition in preschoolers and might affect other EFs, such as set-shifting and visuospatial working memory.

## Introduction

Playing music is one of the most challenging tasks for the human brain because it involves many cognitive processes simultaneously. Most of these processes require cognitive control, which is summarized into a concept known as *executive functions* (EFs). EFs are described as a family of top-down mental processes including goal-directed behavior, planning and problem solving, such as inhibition, set-shifting, and working memory (Diamond, 2013). Since EFs are associated with academic achievement, intelligence, health, and wellbeing, EFs should be promoted, even early in childhood (Diamond, 2013). Music training might be an effective intervention to improve EFs, because making music, such as playing a musical instrument or singing a song, involves several EFs at the same time without focusing on a particular EF. In the last decade, some studies have reported an association between music training and EFs in children (e.g., Degé et al., 2011a). Additionally, musical training has been shown to enhance EFs in children (e.g., Jaschke et al., 2018). In preschoolers, musical training improves inhibition (e.g., Bugos and DeMarie, 2017). However, researchers have not clearly determined which specific components of music training programs are responsible for these beneficial effects on EFs. Components of musical training, which might be crucial for EFs include rhythmic entrainment (Miendlarzewska and Trost, 2014) or melodic encoding. Therefore, in the present study, we aimed to investigate whether different types of music training improved EFs such as inhibition, set-shifting, and working memory in preschoolers compared to a control sports training program. In particular, we were interested in determining whether rhythm-based music training would produce different beneficial effects on EFs than pitch-based music training.

Many cognitive processes are activated in parallel when an individual plays a musical instrument. The musician must read the music and translate the notes into movements (e.g., finger movements when playing the piano). At the same time, the individual is listening to the music and for mistakes. Additionally, the automatic responses must be inhibited when the key is changing and different chromatic signs are played (inhibition). A shift between different dynamics, rhythms, and tempos within one piece of music is often required (set-shifting). Working memory capacities are involved while remembering musical excerpts. Furthermore, the processes will become more complex when an individual is playing with others, because in addition to controlling your own playing with all of these processes, you must adapt your playing to the sound and music of the other players. For example, a musician must shift his/her attention between parts (set-shifting) and inhibit the impulse to play another part while playing the assigned part (inhibition). These processes are only examples of the variety of processes the human brain must perform while an individual plays music. However, all these processes share the requirement for voluntary cognitive control, which involves EFs. According to Diamond (2013) and Miyake et al. (2000) the three core EFs include inhibition, not acting impulsively, but prevent prepotent responses; set-shifting, switching between tasks or mental sets and working memory, monitoring and updating representations. EFs are associated with health, wealth, academic achievement and quality of life (Diamond, 2013), and studies have shown that EFs are trainable in children (for a review see Diamond and Lee, 2011) and adults (e.g., Richmond et al., 2011; Voss et al., 2011). Therefore, an opportunity to foster EFs in early childhood would be important to reduce disparities in EFs with the aim of affecting later academic achievement and well-being. As Diamond and Lee (2011) stated, repeated practice and an increasing level of difficulty is crucial for the successful training of EFs. Hence, musical training, which is associated with practicing regularly and a continuously increasing level of difficulty, might represent a perfect intervention to enhance EFs. Interestingly, and as described above, musical training affects several EFs at the same time.

Correlational research observed positive associations between music lessons and EFs, showing that music lessons are associated with inhibition (Degé et al., 2011a; Joret et al., 2016), set-shifting, selective attention, planning (Degé et al., 2011a) and fluency (Zuk et al., 2014) in children. Regarding preschoolers, Winsler et al. (2011) observed better inhibitory control in children aged 4 years or older who were enrolled in early music classes than their age-matched peers who had never taken early music classes (*d* = 0.28). Moreover, regardless of age, children who are currently taking early music classes outperform children who are not taking early music classes (*d* = 0.41). The authors postulated that early music classes promote children’s motor behaviors *via* guiding their movements with the aid of music. However, since these studies used a correlational design, they do not allow for inferences of causality. If we consider longitudinal studies of the association between music training and EFs, we also find some evidence that music training has the potential to enhance EFs in children. A study by Holochwost et al. (2017) indicated that intensive school-based music training improves performance on tasks assessing set-shifting (*d*s from 0.18 to 0.35), short-term-memory (*d* = 0.25), and inhibition (*d*s from 0.40 to 0.57) in children from grades 1 to 8. Music training occurred daily and consisted of 40 min of instrumental music classes administered in a small group and 40 min of ensemble rehearsal. In this study, the music training group was compared to an untrained control group. Thus, the authors were unable to exclude the possibility that a Hawthorne or a schooling effect influenced their results. Jaschke et al. (2018) conducted another study using a school-based instrumental music training. The authors compared the effects of weekly school-based music classes to school-based visual arts classes on 6-year-olds. The music classes comprised music theory, joint singing, and playing instruments for one or 2 h per week for 2 years. In contrast to the study performed by Holochwost et al. (2017), the lessons were held with the entire class instead of small groups. Children from the music program were divided into two different groups, one group including children who did not have prior music knowledge and another group including children who were receiving private music lessons in addition to the school curriculum. The school-based music classes enhanced some, but not all, measures of EF. Both music groups exhibited improved performance on tests of inhibition and planning and outperformed the visual arts and no-arts control groups after 2 years of training. Regarding visual working memory, the visual arts group outperformed both music groups and the no arts control group. However, as the children were not randomly assigned to the groups, the findings might have been affected by pre-existing differences between groups that were not controlled. According to Roden et al. (2012), school-based music training enhances verbal (*d*s from 0.65 to 1.27), but not visual memory in primary school children compared to natural science training. Children from the music group received weekly 45 min of instrumental music training conducted in a small group with a maximum of five children. In addition, they participated in singing, rhythm and pitch exercises. Another study investigating the effect of music training on working memory showed that 2 years of an extended school-based music curriculum enhanced visual (*η*^2^ = 0.12) and auditory working memory (*η*^2^ = 0.23) in children aged between 9 and 11 years (Degé et al., 2011b). Similar to the study by Holochwost et al. (2017), children participating in the extended music curriculum received intensive training in five to seven music classes per week, including music theory, instrumental instruction and participating in the choir and/or the orchestra. Since the measure for visual working memory also relied on verbal components, the authors were unable to clearly determine if the music training actually affected the visual working memory or whether the training also enhanced the verbal working memory that was also involved in the task. Accordingly, researchers have not clearly determined whether music training has the potential to improve visual working memory. Another reason for this uncertainty is that the studies by Roden et al. (2012) and Degé et al. (2011b) did not randomize the participants; therefore, the results might have been influenced by pre-existing differences in children. In summary, previous studies suggest that school-based music lessons enhance EFs, such as inhibition (Holochwost et al., 2017; Jaschke et al., 2018), set-shifting (Holochwost et al., 2017), planning (Jaschke et al., 2018) and working memory (Degé et al., 2011b; Roden et al., 2012).

Regarding longitudinal studies with younger children, two studies have shown that music training enhances inhibition in preschoolers. In a study by Moreno et al. (2011), children aged 4–6 years were randomly assigned either to a group receiving computerized music listening training or to a group receiving computerized visual arts training five times a week for 45 min over a period of 20 days. Before and after training, all children completed a Go-Nogo task measuring inhibition. Children from the music group exhibited significant improvements from the pre-test to the post-test and outperformed children from the visual arts group ($\eta_{\text{p}}^{2}$ = 0.12). Therefore, the authors concluded that a short-term intense computerized hearing-based music training program improves inhibition in preschoolers. Another study compared preschoolers receiving 45 min music classes twice weekly for a period of 6 weeks in kindergarten to a control group of preschoolers receiving the same amount of LEGO training (Bugos and DeMarie, 2017). In contrast to the findings reported by Moreno et al. (2011), the music training program was a practical music training program involving tasks such as vocal development, improvisation and bimanual gross motor coordination. Children were administered the Matching Familiar Figures Test (MFFT) and the Day/Night Stroop Task before and after training to test inhibition. Based on the results, the scores of all children on both tests improved from before to after training. Moreover, the music group outperformed the LEGO group on the MFFT after training (*d* = 0.99). Hence, the authors concluded that short-term music classes enhance complex inhibition tasks in preschoolers. Since inhibition appears to be the key EF during early childhood (Wiebe et al., 2008), music training likely has no impact on other EFs, such as set-shifting and working memory, in preschoolers. However, the reported studies did not investigate the effect of music training on other EFs. Therefore, researchers have not yet clearly determined whether music training improves inhibition alone or if it has the ability to influence other EFs in preschoolers. Taken together, the current state of knowledge indicates that music training enhances several EFs in children. In preschoolers, the reported studies showed that a short-term, computerized music listening training (Moreno et al., 2011) and a comprehensive practical music training program including vocal development, improvisation and gross motor coordination (Bugos and DeMarie, 2017) enhance inhibition. However, based on these studies, we are unable to conclude that a special component of musical training improves inhibition or whether a combination of different music components improve inhibition in preschoolers.

Most of the studies reported above used instrumental music training programs (e.g., Roden et al., 2012; Holochwost et al., 2017) or a comprehensive music training program (e.g., Bugos and DeMarie, 2017) including various components of music, such as pitch, rhythm, improvisation and bimanual coordination. The instrumental training programs consisted of general instrumental music instruction conducted in a small group (e.g., Roden et al., 2012) or in a class (Jaschke et al., 2018). In some studies, children also received lessons in ensemble playing (Holochwost et al., 2017) or music theory (Jaschke et al., 2018). The music training taught in kindergarten consisted of vocal development exercises, gross motor coordination using different instruments such as drums and xylophones, and improvisation tasks (Bugos and DeMarie, 2017). Moreno et al. (2011) did not administer practical music training in their study, but instead provided computerized hearing-based music training that comprised tasks related to pitch and rhythm. The music training programs varied in the length of lessons, ranging from 45 min per week (Roden et al., 2012) to 80 min per day (Holochwost et al., 2017), and in the duration of the training, ranging from a few weeks (e.g., Moreno et al., 2011; Bugos and DeMarie, 2017) to 2 years (e.g., Jaschke et al., 2018). In summary, substantial variety exists in the music training programs administered in current research. However, all music training programs include a mixture of components related to pitch and rhythm. Therefore, none of these studies provides information about possible differential effects of the single components on EFs. Only one study by Patscheke et al. (2018) examined the effects of a pitch-based music training compared to a rhythm-based music training on phonological awareness, but not EFs, in preschoolers. The pitch-based music training, but not the rhythm-based music training, influenced phonological awareness skills. This study proposed the hypothesis that different components of music training exert different effects on cognitive abilities in general. Therefore, single components of a music training program, such as rhythm and pitch training, might also exert different effects on EFs. Furthermore, Miendlarzewska and Trost (2014) suggested that rhythmic entrainment in particular might be one important component of a musical training program that would affect cognitive processes, such as EFs. The term *entrainment* is defined as the synchronization of at least two independent rhythmic processes (Clayton et al., 2004). In music, a simple example is clapping or tapping to the beat of a song. The ability to synchronize clapping or tapping to a specific rhythm requires the auditory perception of the rhythm, coordination of movements and sensorimotor integration. According to a study by Krause et al. (2010), musicians appear to exhibit increased connectivity in a brain network involving the premotor cortex, posterior parietal cortex and thalamus. These areas are also associated with attentional processes and motor planning (Coull, 2004), prompting the hypothesis that rhythmic entrainment plays a key role in the effect of music training on the development of EFs (Miendlarzewska and Trost, 2014). Thus, musical rhythm training in a group setting potentially represents an appropriate intervention to promote rhythmic entrainment abilities, because the players must automatically synchronize to each other in a natural setting.

We conducted the present study to investigate the differential effects of pitch-based music training compared to a rhythm-based music training on EFs in German preschoolers. Previous studies have already reported an effect of music training on performance on different tests of inhibition (Moreno et al., 2011; Bugos and DeMarie, 2017). However, researchers have not determined whether a special aspect of music training leads to the reported effects or if different musical aspects train various EFs. Miendlarzewska and Trost (2014) proposed that rhythmic entrainment is one important factor leading to cognitive enhancement. Moreover, the study by Patscheke et al. (2018) indicated different effects of various components of a music training program, such as pitch training and rhythm training, on phonological awareness. Therefore, we decided to use the same training program as Patscheke et al. (2018) to explore possible differences in the effects of a pitch-based music training compared to a rhythm-based music training on EFs in preschoolers. In addition to the music training groups, we added a third group receiving sports training as a no-music training control group. Based on the findings reported by Miendlarzewska and Trost (2014), we predicted a greater improvement in inhibition in children from the rhythm group, because the rhythm training focused on rhythmic perception and production, which are strongly associated with rhythmic entrainment. Inhibition appears to be the most important EF during early childhood (Wiebe et al., 2008). Since previous studies showed an improvement in inhibition in preschoolers receiving music training, we were specifically interested in investigating possible differences in the effects of pitch-based music training compared to rhythm-based music training on inhibition. Nevertheless, we decided to test the effects of the training programs on the other core EFs, including set-shifting and working memory, because studies with older children suggest that music training affects several EFs (e.g., Holochwost et al., 2017). The reported studies did not examine whether music training affected other EFs or inhibition alone in preschoolers.

## Materials and Methods

### Participants

At the pre-test, the sample consisted of 95 preschoolers (57 females) aged 5–6 years (*M* = 5.7 years, *SD* = 0.3 years). Participants were recruited from different kindergartens^1^ in the City of Giessen, Germany and the surrounding area. Participants were randomly assigned either to a music group receiving pitch training (*n* = 33), a music group receiving rhythm training (*n* = 33), or a control group receiving sports training (*n* = 30). We determined the inclusion criteria for our analyses to a training participation rate of at least 66%. The remaining sample included in the analyses comprised 76 children (see below for details). None of these children received music lessons or participated in a music group such as a choir or an ensemble. Some children (16%) had taken early music education for an average of *M* = 14.4 months (*SD* = 7.6 months). The socioeconomic status was assessed based on the parents’ education levels and the monthly family income. The parents of most children (65%) did not have a university degree, one parent of 16% of the children had a university degree, and both parents of 11% of the children attained a university degree. Some parents did not provide information about their educational level (11.6%). The monthly family income in the sample ranged from less than 1,000€ (8%) to more than 5,000€ (3%). Most families reported a monthly income ranging from 1,000€ and 2,000€ (28%) or 2,000€ and 3,000€ (20%). Some parents (18%) did not provide details about their monthly income. We did not detect group differences in demographic variables. For additional details, please see Table 1.

**Table 1:** Inferential statistics for group comparisons of control variables.

Variable	Pitch *M* (*SD*)	Rhythm *M* (*SD*)	Sports *M* (*SD*)	*df*	Statistical value	*p*
Gender	17 F/10 M	18 F/8 M	11 F/12 M	2, *n* = 76	*χ*^2^ = 2.44	0.295
Age^1^	67.44 (3.49)	68.77 (3.43)	69.43 (3.75)	2, 73	*F* = 2.06	0.134
IQ	99.93 (11.56)	99.08 (8.39)	98.13 (12.62)	2, 73	*F* = 0.17	0.846
Early music education^1^	3.36 (7.40)	1.50 (5.38)	1.14 (3.61)	2, 67	*F* = 1.01	0.368
Participation in training^2^	84.19 (9.42)	82.23 (9.74)	80.00 (8.37)	2, 73	*F* = 1.28	0.285
Enjoyment of training	4.43 (0.55)	4.34 (0.95)	4.56 (0.68)	2, 59	*F* = 0.49	0.617
Parental education	0.6 (0.82)	0.39 (0.72)	0.19 (0.40)	2, 66	*F* = 2.06	0.135
Family income	3.48 (1.41)	2.60 (1.27)	2.68 (0.86)	2, 59	*F* = 3.40	0.04*^a^
Extraversion scale BFI	3.93 (0.67)	4.02 (0.69)	3.90 (0.76)	2, 69	*F* = 0.32	0.725
Agreeableness scale BFI	3.72 (0.40)	3.58 (0.52)	3.59 (0.58)	2, 69	*F* = 0.58	0.560
Conscientiousness scale BFI	3.47 (0.67)	3.41 (0.73)	3.58 (0.80)	2, 69	*F* = 0.30	0.740
Neuroticism scale BFI	2.48 (0.51)	2.53 (0.62)	2.65 (0.54)	2, 69	*F* = 0.54	0.588
Openness scale BFI	3.75 (0.61)	3.66 (0.40)	3.70 (0.46)	2, 69	*F* = 0.21	0.811

^1^ In Months, ^2^ in %, ^a^*post hoc* analyses did not reach significance. *p < 0.05).

### Materials

#### Training Programs

Preschoolers were trained for 20 min three times a week for a period of 20 weeks. Trained research assistants conducted all sessions. Training programs were based on a manual. Every week, the research assistants and the supervisor of the study met to prepare and practice the training session for the following week to ensure that every training session was implemented in the same way. A typical training session on 1 day consisted of two to four different tasks lasting for a total of 20 min. The training sessions were implemented as described in the study by Patscheke et al. (2018) and based on a well-established early music education program designed by Nykrin et al. (2007).

Rhythm training focused on rhythmic exercises, including meter execution, perception, imitation and production of different rhythms. The exercises were conducted using sound gestures (e.g., clapping and stomping) and different Orff instruments (e.g., taborets, claves, and maracas). Exercises in meter execution included the synchronization of body movements to a given meter, dancing and playing rhythms with percussion instruments. Typical perception and imitation tasks consisted of imitating rhythms using rhythm language (ta-a-a-a, ta-a, ta, and titi) or percussion instruments.

Pitch training focused on discriminating sounds, intonation, sound production, and joint singing. Typical exercises for discriminating sounds were listening to different musical instruments or sounds from a CD and then naming or pointing to the instrument on a picture. Intonation was trained with the call and response method, as the teacher sang intervals or short melodies and the children subsequently repeated the intervals or melodies. Pitch discrimination was trained by listening to different tones from the mallet instrument and indicating which tones were higher or lower or by listening two different voices from a CD and indicating which was higher or lower. In pitch training, we did not use any percussion instruments or sound gestures for rhythmic accompaniment. Conversely, in rhythm training, we did not use melodic instruments or sing any melodies. Nevertheless, some overlap occurred between the training programs, because rhythm is to some extent also connected to prosodic features and song cannot be sung without using a certain rhythm. However, we reduced the overlap between trainings as much as possible.

The manual for sports training was the same as used by Patscheke et al. (2018) and comprised different exercises to practice body perception, motor skills and body coordination by supporting balance, physical strength, endurance and relaxation. Similar to the music training programs, a typical session included two to four different tasks for a total of 20 min. The children were asked to perform different exercises for body perception (e.g., exercises from yoga), balance (e.g., balancing objects on different parts of the body), motor skills (e.g., throwing balls into a box from a near or far distance) and different coordination and cooperation games in a group (e.g., walking altogether as a caterpillar). The sports training program was based on *Yoga and active games for kids* by Dunemann-Gulde (2005).

All training programs involved the same level of engagement of the children, and each trainer conducted classes in each training program to exclude trainer bias.

### Measures

#### Control Variables

Demographic variables such as socioeconomic status (assessed based on parental education and family income) and the musical background of the children (e.g., if the child had participated in courses for early music education) were assessed using a questionnaire. The parental education level was coded as follows: 0 = no parents holding a university degree, 1 = one parent holding a university degree, and 2 = two parents holding a university degree. Family income was assessed with a six-point scale ranging from less than 1,000*€* per month to more than 5,000€ per month. Early music education was assessed in months. Furthermore, parents completed the German version of the Big Five Inventory (BFI; Rammstedt and Danner, 2017) to assess the personality of each of their participating children. The BFI is a questionnaire comprising 45 items designed to measure the Big Five factors of personality (openness, agreeableness, extraversion, conscientiousness and neuroticism). Parents were asked to rate the extent to which they would agree with specific statements (e.g., “I see my child as someone who is talkative”) on a 5-point Likert scale. As described in the study by Rammstedt and Danner (2017), we calculated the means for each of the personality factors. Reversed items were recoded. Higher scores indicate a stronger characteristic of the personality factor. The five scales of the German BFI show an internal consistency of *α* = 0.74 to *α* = 0.86. The test-retest reliability for the scales ranges from *r* = 0.78 to *r* = 0.93. We administered the revised version of the Culture Fair Test (CFT 1-R) created by Weiß and Osterland (2012) to assess fluid intelligence. The CFT 1-R is divided into two parts including three subtests. The first part measures figural perception and processing speed with the subtests *substitutions, mazes* and *similarities*. The second part includes the subtests *continuing series*, *classifications* and *matrices* to measure figural reasoning. All subtests contain 15 items that must be processed in a certain time. Research assistants explained examples of each subtest according to the manual before the subtests began. The test provides age-corrected standard values for children aged from 5.3 to 9.11 years. The test-retest-reliability is *r* = 0.88 for the first part, *r* = 0.94 for the second part and *r* = 0.95 for the whole test. We assessed children’s enjoyment of participating in the trainings to control for potential biases concerning their motivation and willingness. Every 5 weeks of the trainings, children rated on a 5-point-Likert scale how much they enjoyed participating in the training. The scale ranged from 1 = not a bit to 5 = very much and was presented to the children *via* smileys (1 = very sad smiley; 5 = very happy smiley; 3 = neutral smiley). For each child, we generated an average score of the four measurements.

#### Dependent Variables

The EF measures inhibition, set-shifting and working memory served as our dependent variables and were assessed before and after training. Inhibition was measured using the subtest “statue” from the NEPSY-II (Korkman et al., 2007), a developmental neuropsychological assessment for children. The test is designed to assess motor persistence and inhibition. During the test, the child was asked to stay in a particular position with his/her eyes closed for 75 s. The child was asked not to respond to any sound distractor during this time. The experimenter made different noises as distractors at certain times (e.g., after 20 s the experimenter drops a pencil onto the table). At every 5-s interval, the experimenter recorded if the child committed any error by opening his/her eyes, moving the body or responding verbally to the sound or if the child had no errors. For every 5-s interval, the child received two points if no errors were recorded, one point if two errors were recorded or zero points if more than two errors were recorded during that interval. We added the points for all time intervals as the outcome measure and compared them with age-corrected norms, which are provided for children aged from 3 to 6 years. The test-retest-reliability of this subtest is *r* = 0.88 for children aged from 5 to 6 years.

Set-shifting was assessed with the Dimensional Change Card Sort (DCCS; Zelazo, 2006). We administered both the standard and more challenging border versions. In the standard version, children were presented two target cards (a blue bunny and a red boat) and invited to sort the test cards (red bunnies and blue boats) according to one dimension. In the pre-shift phase, children were asked to sort all cards according to the dimension “color,” and were required to place a red card to the side of the red boat and a blue card to the side of the blue bunny. After 6 trials, the experimenter interrupted the game and changed the dimension to “shape,” and thus the child sorted cards with the bunny shape to the blue bunny and cards with the boat shape to the red boat (post-shift). Both the pre-shift and post-shift phases comprised 6 trials, with a total of 12 trials for the standard version. After completing the standard version of the test, we administered the more challenging extended version, which combined both dimensions from the standard version. In the extension, the child was asked to sort cards with a black frame according to the dimension “color” and cards without a frame according to the dimension “shape.” Similar to the standard version, the extended version also consisted of 12 trials. In both versions, we repeated the instructions after every trial to ensure the lowest demands on working memory as possible. In the pre-shift and post-shift phases, children were required to sort at least five of six cards correctly to pass. In the extended version, children were required to sort at least 9 of 12 cards correctly to pass. We used the scoring system suggested by Zelazo (2006): children who failed the pre-shift phase received a “0,” children who passed the pre-shift phase received a “1,” children who passed the post-shift phase but failed the extended version received a “2” and children who passed both the standard and extended versions received a “3.” The DCCS shows an intra-class correlation (*ICC*) of 0.94 for the standard version and *ICC* = 0.90 for the extended version (Beck et al., 2011).

We measured visual-spatial working memory by administering the subtests Matrix Span Test (for visual cache) and Corsi Block Test (for inner scribe) from the Working Memory Test Battery for 5- to 12-year-old children (“Arbeitsgedächtnistestbatterie 5–12,” AGTB 5–12) described by Hasselhorn et al. (2012). According to Logie (1995), visual working memory is divided into two subcompartments: the visual cache and the inner scribe. The visual cache is responsible for storing information about shape and color, whereas the inner scribe analyses information about location and movement. Both tests consisted of a standardized computerized introduction with two practice trials and 10 test trials. The difficulty level at the beginning of the test trials depended on the child’s age. For example, 6-year-old children started at a more difficult level than 5-year-old children. Both subtests used an adaptive procedure that increased the difficulty for correct responses and decreased the difficulty for incorrect responses (described in detail below). During the test trials, children were not informed about their test performance. The AGTB provides age-corrected standard values for children aged from 5 to 12 years. The Matrix Span Test measures the memory of static visual patterns. Children were presented a 4 × 4 matrix composed of white and black squares on a touchscreen for 4 s. After the matrix had disappeared, children were shown a new matrix with white squares only and asked to tag the squares that were black in the previous image. If the child responded correctly in two consecutive trials, the number of the black squares increased by one (to a maximum of nine black squares). On the other hand, if the child responded incorrectly in two consecutive trials, the number of black squares was reduced by one (to a minimum of two black squares). The Corsi Block Test assessed the memory of dynamic changes in spatial locations. In this test, children were asked to memorize the path of a yellow smiley face that moved between nine white squares displayed on a gray background on the touchscreen. After the full path of the smiley face was shown, children recorded their response by touching the order of the squares in which the smiley face had moved. If the child responded correctly in two trials, the path of the smiley face was increased by one square (to a maximum of nine squares). If the child responded incorrectly in two trials, the path became one square shorter (to a minimum of two squares). The test-retest-reliabilities for both subtests are *r* = 0.66 for children aged from 5 to 8 years.

### Procedure

The experiments were conducted in accordance with ethical guidelines of the ethics committee of the Faculty of Psychology and Sports Science at Giessen University (application number 2015-0001). Prior to pre-tests, parents were provided information sheets about the aims and contents of the study and informed consent was obtained for each participant. All test sessions were conducted as single sessions in a separate room in kindergarten. Trained research assistants from the department, who were at all times blinded to the conditions and the hypotheses, administered test sessions. We started test sessions with the DCCS, followed by the statue test and the tests of visual working memory. The CFT 1-R was administered in an extra session on the same day after an appropriate break or on a consecutive day. After the pre-tests, children were randomly assigned to one of the music groups or to the sports group. All training programs were conducted in small groups of 5–8 children for 20 weeks. Groups participated in the designated training program three times a week for 20 min each. After the training phase was complete, post-tests of the dependent measures were immediately administered using the same method described for pre-test. When post-tests were complete, each child received a small present and a certificate as a reward for participating in the study.

## Results

All analyses were computed using *IBM*^®^ *SPSS*^®^ *Statistics 25*. First, we analyzed the dropout rate to ensure that the children who dropped out did not differ significantly from the remaining sample. Children with a training participation rate of less than 66% were excluded and counted as a dropout. Of the 95 children enrolled, 19 did not meet this inclusion criterion, and thus the dropout-rate in our sample was 18.1%. Major reasons for the low training participation rate of these children were illness and winter holidays. Based on our analyses, these children did not significantly differ from the included sample in terms of gender, $\chi_{\left(1\right)}^{2}$ = 0.04, *p* = 0.834, age, *t*_(93)_ = 0.31, *p* = 0.757, parental education *t*_(82)_ = −0.86, *p* = 0.392, family income, *t*_(75)_ = −1.18, *p* = 0.243 and IQ, *t*_(93)_ = −0.05, *p* = 0.960. A power analysis using G*Power (Erdfelder et al., 1996) suggested that a total sample size of 66 participants was sufficient to ascertain small to medium effects (*f* = 0.25) in a repeated-measures within-between design (α: 0.05, power (1-*β*): 0.95, correlations between repeated measures: *r* = 0.50).

### Preliminary Analyses

We compared differences in control variables between the training groups. As shown in Table 1, significant differences in gender, age, IQ, early music education, training participation, enjoyment of the training, parental education and personality were not observed between the training groups. A significant difference in family income was identified, indicating that families from the pitch group had the highest income (*M* = 3.48, *SD* = 1.41), followed by the sports group (*M* = 2.68, *SD* = 0.89) and the rhythm group (*M* = 2.60, *SD* = 1.27). However, Bonferroni-adjusted *post hoc* analyses did not reveal significant (*p* < 0.05) differences between the pitch and rhythm groups [95% CI (−0.05, 1.8)], between the pitch and sports groups [95% CI (−0.14, 1.73)] or between the rhythm and sports groups [95% CI (−0.1.05, 0.88)]. We performed ANOVAs for the EFs measured in the pre-test to exclude the possibility of any pre-existing differences in EFs between groups. No differences in inhibition, *F*_(2,73)_ = 0.45, *p* = 0.64, set-shifting *F*_(2,73)_ = 1.26, *p* = 0.29, and working memory *F*_(2,73)_ = 1.36, *p* = 0.26 (Matrix Span), *F*_(2,73)_ = 0.43, *p* = 0.65 (Corsi Block) were observed between groups. Taken together, the preliminary analyses did not reveal significant systematic differences in control variables or dependent measures at the pre-test.

### Main Analyses

We performed repeated measures ANOVAs with the training group as the between-subjects factor and time as the within-subjects factor for the tests of inhibition, set-shifting, and working memory. Mean values and standard deviations for the dependent variables are presented in Table 2.

**Table 2:** Means and standard deviations of dependent variables for treatment groups and the control group at the pre-test (T0) and post-test (T1).

Variable	Pitch *M* (*SD*)		Rhythm *M* (*SD*)		Sports *M* (*SD*)	
	T0	T1	T0	T1	T0	T1
**Inhibition**						
Statue test	11.11 (2.14)	11.81 (2.20)	10.46 (2.47)	12.17 (1.93)	11.00 (2.27)	10.86 (2.26)
**Set-shifting**						
DCCS	2.07 (0.55)	2.41 (0.57)	1.96 (0.66)	2.50 (0.51)	1.83 (0.39)	2.13 (0.55)
**Working memory**						
Matrix span	46.81 (9.12)	51.11 (9.16)	50.27 (7.50)	48.92 (10.96)	49.87 (8.19)	53.74 (11.30)
Corsi block	46.78 (8.51)	50.00 (6.58)	44.88 (7.25)	50.65 (6.14)	46.78 (9.66)	47.57 (7.62)

**Figure 1:**
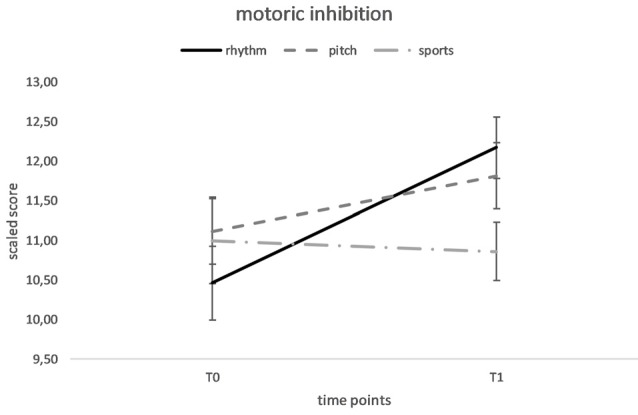
Mean performance of motoric inhibition at baseline (T0) and after 20 weeks of rhythm, pitch or sports training (T1). Error flags indicate Standard Errors of Means (SEM).

For inhibition, the repeated measures ANOVA indicated a significant main effect of time, *F*_(1,73)_ = 9.03, *p* = 0.004, *d*_RM_ = 0.33, showing an overall improvement from the pre-test (*M* = 10.86, *SD* = 2.28) to the post-test (*M* = 11.64, *SD* = 2.02), as well as a significant training group × time interaction *F*_(2,71)_ = 4.18, *p* = 0.019, *d* = 0.70. The main effect of group was nonsignificant, *F*_(1,73)_ = 0.52, *p* = 0.598. We analyzed the training group × time interaction by performing a 2 (time) × 2 (training group) ANOVA and observed a significant training group × time interaction when comparing the rhythm and sports groups, *F*_(1,42)_ = 7.14, *p* = 0.011, *d* = 0.84. We did not observe significant interactions with time when the pitch and sports groups were compared, *F*_(1,47)_ = 4.28, *p* = 0.124 or when the pitch and rhythm groups were compared, *F*_(1,49)_ = 6.41, *p* = 0.12. Paired *t*-tests showed a significant improvement from the pre-test to post-test for the rhythm group, *t*_(23)_ = −3.21, *p* = 0.004, *d*_RM_ = 0.56, whereas the results for the pitch group, *t*_(27)_ = −1.94, *p* = 0.062, and the sports group, *t*_(21)_ = 0.34, *p* = 0.734 were nonsignificant. Independent *t-*tests did not reveal differences between the rhythm and the sports group at the pre-test, *t*_(47)_ = −0.27, *p* = 0.79, but indicated a significant difference between the rhythm and the sports group at the post-test, *t*_(42)_ = 2.34, *p* = 0.02, *d*_corr_ = 0.82 (see Figure 1).

**Figure 2:**
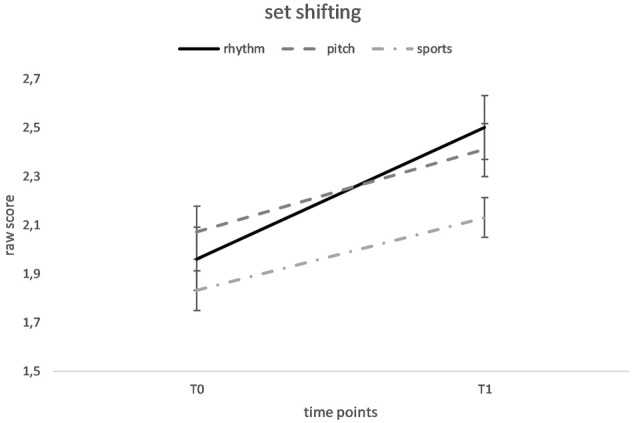
Mean performance of set-shifting Dimensional Change Card Sort (DCCS) at baseline (T0) and after 20 weeks of rhythm, pitch or sports training (T1). Error flags indicate SEM.

Regarding set-shifting, a repeated measures ANOVA revealed a significant main effect of time, *F*_(1,73)_ = 21.58, *p* < 0.001, *d*_RM_ = 0.55, showing an overall improvement from the pre-test (*M* = 1.96, *SD* = 0.55) to the post-test (*M* = 2.35, *SD* = 0.56), as well as a significant main effect of the training group, *F*_(2,73)_ = 3.23, *p* = 0.045, *d* = 0.59. However, Bonferroni-adjusted *post hoc* group comparisons between the rhythm and sports groups [95% CI (−0.03, 0.54)], the rhythm and pitch groups [95% CI (−0.28, 0.26)] and the pitch and sports groups [95% CI (−0.02, 0.55)] were nonsignificant (all *p*-values > 0.05). Furthermore, the training group × time interaction was nonsignificant, *F*_(2,73)_ = 0.77, *p* = 0.47 (see Figure 2).

**Figure 3:**
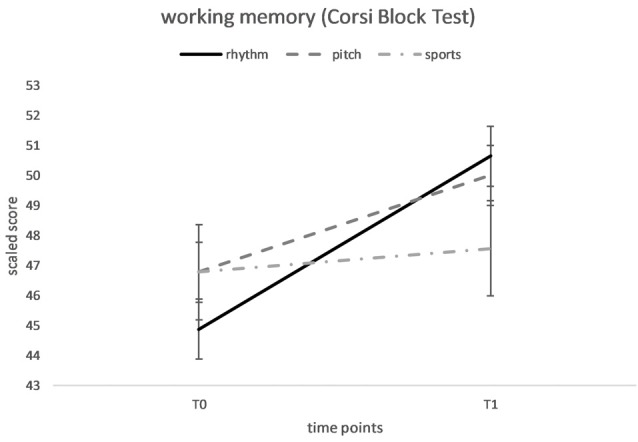
Mean performance of visuospatial working memory (Corsi Block Test) at baseline (T0) and after 20 weeks of rhythm, pitch or sports training (T1). Error flags indicate SEM.

Repeated measures ANOVAs for working memory did not reveal a significant main effect of time on the Matrix Span result, *F*_(1,73)_ = 3.55, *p* = 0.064, or a significant main effect of the training group *F*_(2,71)_ = 0.88, *p* = 0.421. The training group × time interaction was also nonsignificant, *F*_(2,71)_ = 2.31, *p* = 0.11. For the Corsi Block Test, we observed a significant main effect of time, *F*_(1,73)_ = 10.67, *p* = 0.002, *d*_RM_ = 0.35, showing an overall improvement from the pre-test (*M* = 46.13, *SD* = 8.42) to the post-test (*M* = 49.49, *SD* = 6.80), but a significant main effect of the training group was not detected, *F*_(2,71)_ = 0.23, *p* = 0.795. The training group × time interaction was also nonsignificant, *F*_(2,71)_ = 2.02, *p* = 0.14 (see Figure 3).

## Discussion

We explored differences in the effects of rhythm-based music training and pitch-based music training on EFs in preschoolers. Based on our results, 20 weeks of rhythm training enhanced motor inhibition, but not pitch-based music training and sports training. The effect of the rhythm training program corroborates the hypothesis that rhythmic entrainment represents an important underlying mechanism of music training that supports cognitive function (Miendlarzewska and Trost, 2014). Moreover, since the rhythm training program required a high level of motor coordination we postulated that the motor component of the rhythm training program specifically influenced preschoolers’ inhibition skills.

Our study confirms results from previous studies by showing an effect of music lessons on inhibition in school-children (Holochwost et al., 2017; Jaschke et al., 2018) and preschoolers (Bugos and DeMarie, 2017). However, most of these studies had some methodological issues, such as the lack of an active control group (Holochwost et al., 2017) or a nonrandomized sample (Jaschke et al., 2018), and thus these results might also be attributed to a general training effect or to some non-controlled influencing factors. Accordingly, only our study and the study by Bugos and DeMarie (2017) actually allow for interpretations of causality because the children were randomly assigned to the different training programs. We expand the results of the study by Bugos and DeMarie (2017) by showing that rhythm training particularly appeared to improve inhibition in preschoolers. The rhythmic synchronization while drumming and the precise timing required while producing rhythms and rhythmical movements to music might have improved the inhibition abilities. Since all these rhythmic activities required children’s motor control, it is likely that rhythm-guided motor control, in general, improves motor inhibition in children. Therefore, our findings support the idea of Winsler et al. (2011), who suggested that modulating children’s movements with the aid of music may be beneficial for motor behavior and self-regulation. As shown in the present study, rhythm-guided motor control was implemented in the rhythm training program at a high level and improved motor inhibition, whereas tasks focusing on pitch with less motor involvement did not affect motor inhibition skills in preschoolers. Moreover, the sports training program, which also required a high level of motor control, did not show an effect on motoric inhibition. Thus, we can conclude that rhythm-guided motor control in particular and not motor control alone improved inhibition skills in preschoolers.

Regarding set-shifting, the results showed an overall improvement from the pre-test to the post-test, but the training programs did not exert significantly different effects, since the training group × time interaction was nonsignificant. Hence, we were unable to generate statistically verified conclusions about a special effect of a single training program. However, the descriptive data (see Figure 2) appear to show greater improvements in the music groups than in the sports group, with the rhythm group attaining the greatest benefit. Therefore, our results are consistent with the findings from the study by Holochwost et al. (2017), which showed that music training enhanced set-shifting abilities in older children. However, since we did not identify a significant group by time interaction and all groups exhibited significant improvements in set-shifting from the pre-test to the post-test, further studies are required to corroborate this finding.

Regarding visuospatial working memory, we did not find any significant effect of the training programs on the Matrix Span Test, which assesses the visual cache (memory of static visual patterns), but an overall improvement was observed on the Corsi Block Test, which measures the inner scribe (memory of a dynamic series of spatial locations), from the pre-test to the post-test. Since the training group × time interaction was nonsignificant, statistical confirmation of an effect of a single training program was not obtained. However, based on the descriptive data (see Figure 3), the music groups exhibited improvements in the inner scribe component, with the rhythm group showing the greatest improvement from the pre-test to the post-test, whereas the sports group recorded similar performances on the pre- and post-tests. A potential explanation is an effect of the rhythm-based music training on the inner scribe component, but due to less power and high variances, we did not observe a statistically significant training group × time interaction. Interpreting the descriptive data, our study also confirms the findings reported by Holochwost et al. (2017) of an association between music training and visuospatial working memory in older children to some extent. The improvement on the Corsi Block Test but not the Matrix Span Test might indicate that the music training program did not improve the memory capacity, but improved the processing of visual information. This hypothesis is consistent with the findings from studies assessing verbal memory, showing that music lessons do not enhance verbal storage itself but improve the articulatory rehearsal (Franklin et al., 2008; Degé and Schwarzer, 2017). Therefore, a similar situation might occur for the visual working memory such that the music training program did not affect the visual storage but the processing of information in the visual memory. However, as stated above, we did not find a significant group by time interaction and must interpret these results with caution.

Our results confirm the hypothesis that different components of a music training program exert different effects on cognitive abilities in general. Furthermore, the study by Patscheke et al. (2018) revealed that pitch training, but not rhythm training, affected phonological awareness. Since we administered the same program for the pitch and the rhythm training, a comparison of the results suggests that the training programs exert different effects on cognitive abilities. Consequently, the rhythmic motor aspect of a music training program might improve general cognitive abilities, such as EFs, and intonation and pitch are strongly correlated with verbal abilities such as phonological awareness.

In summary, a rhythm-based music training program enhanced inhibition in preschoolers. As mentioned above, the motor component of the rhythm training program and rhythmic synchronization might be the components of a music training program specifically affect EF. Furthermore, our results show a descriptive tendency that music training affects other EFs, such as set-shifting and visual-spatial working memory, in preschoolers as well. However, since we were unable to statistically confirm this hypothesis, further studies are required to investigate these effects. As inhibition appears to be a key factor in the development of EFs in early childhood, the effect of the rhythm-based music training program on inhibition is an important finding. Since EFs exert far-reaching effects on crucial developmental functions, such as intelligence, academic achievement, wellbeing and health (Diamond, 2013), EFs should be enhanced, even early in childhood. As the present study suggests that regular music training, particularly training focusing on rhythmic elements, improves inhibition, one recommendation is to implement elements of a rhythm-based music training program in already established EF training programs. Furthermore, the development of a music-based EF training program that can be integrated in the daily lives kindergarteners might produce promising effects. The implementation of a music-based EF training program in kindergarten might allow all children to improve their EFs in a manner independent of social contexts to ensure equal opportunities to benefit from the program. Nevertheless, further studies are needed to examine the applicability and the effectiveness of these training programs.

## Limitations and Future Directions

We explored differences in the effects of music training programs on EFs in preschoolers. The results showed an effect of rhythm-based music training on motor inhibition. One limitation of the present study is that our music training programs had some overlapping aspects, as rhythm training cannot be completely separated from pitch training, since melodies and songs are always based on rhythm. However, the main difference between the training programs was that the rhythm training program focused on the music-guided motor component by perceiving rhythms with the whole body and producing rhythms using sound gestures, percussion instruments or rhythmical body movements. In contrast, the pitch training program concentrated on pitch and intonation without any rhythmic motor action. Regarding the sample size, the present study already exhibited an acceptable power, particularly if we consider the costs and efforts associated with the high training intensity and the organization required to work with many different kindergartens. Nevertheless, future studies with larger sample sizes should be conducted to support the generalizability of our findings. Although the power analysis revealed a sufficient sample size in the present study, a larger sample is preferred and might even reveal smaller effects that could have been overlooked in our study. As rhythm training appears to be an important factor influencing inhibition in preschoolers, an investigation of the effect of dancing on EF would also be interesting. Similar to music training, dancing relies on rhythm and meter and includes the musical component as the dancer executes movements to the rhythm of music. Furthermore, similar to our rhythm training program, dancing concentrates on music-guided motor control and rhythmic entrainment, and thus dancing might exert a similar effect on EFs.

## Conclusion

Taken together, music training influences inhibition in preschoolers and might affect other EFs, such as set-shifting and working memory. This study is the first to investigate the different effects of distinct music training programs on several EFs in this age group. In particular, a rhythm-based music training program affects inhibition in preschoolers. In contrast to some previous studies, our results allow for causal interpretations, since we used a randomized controlled design. Therefore, the motor component of rhythm training and rhythmic entrainment represent important components of a music training program that is designed to improve EFs. Further studies examining different age groups and larger sample sizes are required to confirm our findings. Moreover, we recommend a study examining the effect of dancing on EFs, because dancing relies on rhythmic motor movements as well. Therefore, dancing might exert a similar effect on EF. Finally, music-based rhythm training administered in a small group appears to be a suitable intervention to improve inhibition in preschoolers.

## Data Availability

The datasets generated for this study are available on request to the corresponding author.

## Ethics Statement

### Human Subject Research

The studies involving human participants were reviewed and approved by Lokale-Ethikkommission Fachbereich 06 (LEK-FB06), Justus-Liebig-University of Giessen, Germany. Written informed consent to participate in this study was provided by the participants’ legal guardian/next of kin.

## Author Contributions

FD and GS conceived the study. FD designed the study and obtained funding through a DFG grant. UF conducted the experiments and analyzed the data. UF, GS and FD wrote the manuscript.

## Conflict of Interest Statement

The authors declare that the research was conducted in the absence of any commercial or financial relationships that could be construed as a potential conflict of interest.

## Footnotes

^1^In Germany, children aged between 3 and 6 years attend kindergarten. Kindergarten is not compulsory and kindergartens do not have a consistent curriculum. In the last year of kindergarten, when children are aged between 5 and 6 years, they receive some preschool classes. However, children are playing, drawing or doing handicrafts for most of the time in kindergarten.
